# Cultivation and Transcriptional Analysis of a Canonical *Nitrospira* Under Stable Growth Conditions

**DOI:** 10.3389/fmicb.2019.01325

**Published:** 2019-06-26

**Authors:** Aniela B. Mundinger, Christopher E. Lawson, Mike S. M. Jetten, Hanna Koch, Sebastian Lücker

**Affiliations:** ^1^Department of Microbiology, Institute for Water and Wetland Research, Radboud University, Nijmegen, Netherlands; ^2^Department of Civil and Environmental Engineering, University of Wisconsin–Madison, Madison, WI, United States

**Keywords:** nitrification, *Nitrospira*, transcriptomics, continuous stirred tank reactor, nitrite oxidoreductase

## Abstract

Nitrite-oxidizing bacteria (NOB) are vital players in the global nitrogen cycle that convert nitrite to nitrate during the second step of nitrification. Within this functional guild, members of the genus *Nitrospira* are most widespread, phylogenetically diverse, and physiologically versatile, and they drive nitrite oxidation in many natural and engineered ecosystems. Despite their ecological and biotechnological importance, our understanding of their energy metabolism is still limited. A major bottleneck for a detailed biochemical characterization of *Nitrospira* is biomass production, since they are slow-growing and fastidious microorganisms. In this study, we cultivated *Nitrospira moscoviensis* under nitrite-oxidizing conditions in a continuous stirred tank reactor (CSTR) system. This cultivation setup enabled accurate control of physicochemical parameters and avoided fluctuating levels of their energy substrate nitrite, thus ensuring constant growth conditions and furthermore allowing continuous biomass harvesting. Transcriptomic analyses under these conditions supported the predicted core metabolism of *N. moscoviensis*, including expression of all proteins required for carbon fixation via the reductive tricarboxylic acid cycle, assimilatory nitrite reduction, and the complete respiratory chain. Here, simultaneous expression of multiple copies of respiratory complexes I and III suggested functional differentiation. The transcriptome also indicated that the previously assumed membrane-bound nitrite oxidoreductase (NXR), the enzyme catalyzing nitrite oxidation, is formed by three soluble subunits. Overall, the transcriptomic data greatly refined our understanding of the metabolism of *Nitrospira*. Moreover, the application of a CSTR to cultivate *Nitrospira* is an important foundation for future proteomic and biochemical characterizations, which are crucial for a better understanding of these fascinating microorganisms.

## Introduction

The oxidation of nitrite to nitrate by nitrite-oxidizing bacteria (NOB) is a crucial juncture within the microbial driven nitrogen (N) cycle, since nitrite is a substrate for both aerobic and anaerobic processes. Thus, nitrite-oxidizing activity influences whether N compounds are retained in a bioavailable form (i.e., nitrate) or are lost as gaseous emissions from an environment (Bristow et al., 2017). The functional guild of known nitrite oxidizers comprises seven genera (Daims et al., 2016), where members of the genus *Nitrospira* show the highest phylogenetic diversity and widest environmental distribution (Daims et al., 2016). Moreover, *Nitrospira* are the dominant NOB in most non-marine natural environments, even including volcanic soil and hot springs (Marks et al., 2012; Daebeler et al., 2014), but also in marine sponge tissue (Feng et al., 2016), and play a key role in N removal in biological wastewater treatment (Daims et al., 2001).

Recently, genomic insights revealed major differences in the core metabolism between *Nitrospira* and other NOB, including nitrite oxidation and CO_2_ fixation pathways, and the respiratory chain (Lücker et al., 2010; Koch et al., 2015; Ushiki et al., 2018). For example, proteobacterial NOB belonging to the genera *Nitrobacter* and *Nitrococcus*, as well as *Nitrolancea hollandica* of the phylum *Chloroflexi*, use the Calvin–Benson–Bassham cycle for CO_2_ fixation (Starkenburg et al., 2006, 2008b; Sorokin et al., 2012; Füssel et al., 2017), whereas *Nitrospira* employ the oxygen-sensitive reductive tricarboxylic acid (rTCA) cycle (Lücker et al., 2010), possibly reflecting adaptation to microaerophilic conditions (Daims et al., 2016). Moreover, all analyzed *Nitrospira* genomes lack a canonical heme-copper-type terminal oxidase, and instead may utilize a novel cytochrome *bd*-like oxidase, which was proposed based on genomic and transcriptional analyses (Lücker et al., 2010).

The key enzyme of NOB, the nitrite oxidoreductase (NXR), catalyzes the oxidation of nitrite to nitrate and belongs to the DMSO reductase type II family (Lücker et al., 2010). In chemolithoautotrophic nitrite oxidizers, two forms of NXR were identified based on their phylogeny (Lücker et al., 2010) and cellular localization (Spieck et al., 1996, 1998). A periplasmic form of the NXR occurs in *Nitrospira, Nitrospina, Candidatus* Nitromaritima RS, and *Nitrotoga*, whereas *Nitrobacter, Nitrococcus*, and *Nitrolancea* contain a cytoplasmic enzyme complex (reviewed in Daims et al., 2016). The NXR of *Nitrospira* was postulated to be membrane-associated and to consist of a catalytic alpha (NxrA), an electron-transporting beta (NxrB), and a gamma (NxrC) subunit potentially involved in electron transfer to the respiratory chain (Spieck et al., 1996, 1998). In canonical *Nitrospira* genomes, the *nxrAB* genes were always found co-localized and in multiple copies (Lücker et al., 2010; Koch et al., 2015; Ushiki et al., 2018). In contrast to other periplasmic NXR containing NOB, in *Nitrospira*, the candidate genes for NXR gamma subunits are located separately from the *nxrAB* loci (Lücker et al., 2010; Koch et al., 2015; Ushiki et al., 2018). Thus, along with other key metabolic features, the composition of the NXR and the role of the different *nxr* paralogs in *Nitrospira* still await further experimental validation.

Previous biochemical analyses of NOB have mainly focused on *Nitrobacter*, for which biomass can be produced relatively fast and in sufficient amounts (Bock et al., 1983). Thus, the cytoplasmic NXR and the respiratory chain of *Nitrobacter* are better understood than those of *Nitrospira* (e.g., Tanaka et al., 1983; Sundermeyer-Klinger et al., 1984; Meincke et al., 1992; Spieck et al., 1996). However, due to the biotechnological and environmental relevance of *Nitrospira*, a better understanding of their physiology and biochemistry is vital to improve predictions and stability of N-cycling processes. A major prerequisite for such characterization is a method for efficient, stable, and large-scale cultivation. However, *Nitrospira* are notoriously fastidious to culture (Spieck and Lipski, 2010) and the commonly used batch cultivation is laborious and results in low biomass yields. Moreover, fluctuating levels of nitrite and product inhibition from nitrate accumulation are additional drawbacks. An alternative approach is to cultivate NOB in continuous chemostat systems with constant nitrite input and medium exchange as was previously applied for *Nitrobacter winogradskyi* (Mellbye et al., 2015, 2016; Sayavedra-Soto et al., 2015). In addition, continuous-fed bioreactors operated to maintain low residual nitrite levels were successfully applied in the enrichment of *Nitrospira* species (Ushiki et al., 2013; Fujitani et al., 2014).

Here, we established a chemostat system for culturing *Nitrospira moscoviensis* under stable conditions, which allowed us to investigate the transcriptome of *N. moscoviensis* during constant growth. The continuous stirred tank reactor (CSTR) setup enabled accurate control of physicochemical parameters such as dissolved oxygen and substrate loading rates and allowed us to continuously harvest biomass. The transcriptomic data confirmed stable growth as we observed constant gene expression over several generations during a period of 2 weeks. Furthermore, the transcriptomic analysis supports the predicted core metabolism of *Nitrospira* and yielded new insights into, for instance, NXR subunit composition.

## Materials and Methods

### Cultivation

*N. moscoviensis* M-1 was grown in NOB mineral salts medium for lithoautotrophic growth as described in Spieck and Lipski (2010) with the following modifications: CaCO_3_ was replaced with CaCl_2_ ⋅ 2 H_2_O in the same concentration and the following trace element composition was used per liter of medium: 34.4 μg of MnSO_4_ ⋅ 1 H_2_O, 50 μg of H_3_BO_3_, 70 μg of ZnCl_2_, 72.6 μg of Na_2_MoO_4_ ⋅ 2 H_2_O, 20 μg of CuCl_2_ ⋅ 2 H_2_O, 24 μg of NiCl_2_ ⋅ 6 H_2_O, 80 μg of CoCl_2_ ⋅ 6 H_2_O, and 2,000 μg of FeSO_4_ ⋅ 7 H_2_O. Nitrilotriacetic acid was added equimolar to all trace elements as a complexing agent.

*N. moscoviensis* was cultivated in a 7-L glass bioreactor inoculated with an active batch culture. The bioreactor was operated at a 5-L working volume and was equipped with pH, dissolved oxygen, temperature, and level sensors, all connected to a biocontroller (bioreactor and equipment all by Applikon Biotechnology B.V., Schiedam, The Netherlands). The controller setup automatically maintained the pH at 7.7 by constant flushing with Ar/CO_2_ (95%/5% v/v) and buffering with a 1 M KHCO_3_ solution. Dissolved oxygen was kept at 30% by providing filtered air or N_2_ gas through an aeration tube with a porous sparger. The temperature was upheld at 39°C by using a loop-type heat exchanger. CO_2_ levels were controlled manually by increasing Ar/CO_2_ (95%/5% v/v) flow whenever CO_2_ was no longer acidifying the medium as indicated by the use of 1 M KHCO_3_ solution. The culture was constantly stirred at 150 rpm with a marine-style impeller. All cultivation media and solutions were sterilized by autoclaving or sterile filtration prior to use and the reactor was operated aseptically to retain culture purity.

After inoculation, the culture was manually supplied with a final concentration of 3 mM nitrite. Nitrite and nitrate concentrations were checked daily (Nitrite test strips MQuant^^®^^, Merck, Darmstadt, Germany). After the depletion of the initial dose of nitrite, the culture was constantly supplied with fresh medium to avoid nitrate accumulation and to assure sufficient supply of trace elements. For the first 41 days of cultivation, nitrite was manually replenished to 3 mM whenever depleted. Subsequently, the system was switched to a continuous feeding regime by supplying 1 M NaNO_2_ solution with a timer-controlled pump at mass loading rates ranging from 1.3 to 3.9 mM day^-1^. With this setup, the nitrite concentration in the reactor was maintained below 0.1 mM. The culture was monitored for growth and activity by measuring the optical density (OD_600_), total protein content, and nitrite concentrations. Purity of the culture was regularly checked by fluorescence *in situ* hybridization (FISH) and epifluorescence microscopy.

### Analytical Methods

Two-milliliter aliquots of the culture were centrifuged (20,000 × *g*, 15 min) at room temperature (RT). Pellets and supernatant aliquots were stored at -20°C until further analyses. Nitrite concentrations in the supernatant were measured colorimetrically by using the sulfanilamide reaction (Griess, 1879). Protein concentrations of the cell pellets were measured using the Pierce BCA Protein Assay Kit (Thermo Fisher Scientific, Waltham, MA, United States). Cell counts were performed on a fluorescence-activated cell sorter (BD FACSMelody, BD Biosciences, San Jose, CA, United States) using the Trucount kit (BD Biosciences) according to the manufacturer’s protocol. The conversion factor 69,460 ± 4975 cells μg^-1^ protein was determined for the *N. moscoviensis* culture and was used to calculate cell numbers in the reactor.

### Fluorescence *in situ* Hybridization and Microscopy

FISH was performed to check purity of the *N. moscoviensis* culture. Biomass was harvested by centrifugation (20,000 × *g*, RT, 15 min), and the cell pellet was washed twice with 1 × phosphate-buffered saline (PBS). Next, cells were fixed in 4% paraformaldehyde either for 30 min at RT or 3 h on ice (Daims et al., 2005). After fixation, the cells were collected by centrifugation and washed once with 1 × PBS. Fixed biomass samples were stored in 50% ethanol in PBS (v/v) at -20°C. FISH was performed as described in Daims et al. (2005) with a concentration of 35% formamide in the hybridization buffer, with the only modification that hybridizations were performed overnight. Cells were stained with a *Nitrospira*-specific probe mix containing the probes Ntspa662, Ntspa712 (Daims et al., 2001), and Ntspa1151 (Maixner et al., 2006) labeled in Cy3 (Biolegio B.V., Nijmegen, The Netherlands) and the EUB338 probe mix targeting most bacteria labeled in FLUOS (Daims et al., 1999). Probes Ntspa712, Ntspa662, and EUB338I-III were doubly labeled, and the *Nitrospira*-specific probe mix contained the Ntspa662 and Ntspa712 unlabeled competitor probes (Daims et al., 2001). Directly before microscopic analysis, samples were embedded in VectaShield containing 1.5 μg/ml 4′, 6-diamidino-2-phenylindole (Vector Laboratories Inc., Burlingame, CA, United States). The *Nitrospira*-specific signal was compared with the EUB338 probe mix signal using an Axioplan 2 epifluorescence microscope (Carl Zeiss Vision, Breda, The Netherlands) equipped with the AxioVision software Rel. 4.9.1.

### RNA Extraction and Transcriptome Sequencing

Transcriptomic analyses were performed using three technical replicates for each of the three sampling time points. Biomass of 70 ml culture volume was harvested by centrifugation at 20,000 × *g*, 4°C, 10–20 min. RNA was immediately extracted using TRIzol reagent (Thermo Fisher Scientific) following the manufacturer’s protocol with the following modifications: all incubation and centrifugation times were doubled, and RNA was precipitated overnight at -20°C, with subsequent centrifugation at 12,000 × *g*, 4°C, 20–30 min. No heating step for RNA resuspension was included. Residual genomic DNA was digested by DNase I (Thermo Fisher Scientific) treatment following the manufacturer’s protocol. RNA purity was confirmed on an ethidium bromide stained 0.9% agarose gel. The MEGAclear kit (Thermo Fisher Scientific) was used following the manufacturer’s protocol to purify the RNA and remove small RNAs. RNA quantity was controlled using the Qubit RNA HS Assay Kit (Thermo Fisher Scientific). Next, ribosomal RNA was depleted using the MICROBExpress kit (Thermo Fisher Scientific) following the manufacturer’s protocol. Transcriptome libraries were prepared with the Ion Total RNA-Seq Kit v2 (Ion Torrent by Thermo Fisher Scientific). RNA and DNA yield and size distribution were analyzed using a 2100 bioanalyzer (Agilent Technologies, Santa Clara, CA, United States) before and after the rRNA depletion step and after library construction, respectively. Sequencing was performed on an Ion Torrent PGM using the Ion PGM Hi-Q sequencing kit (Ion Torrent by Thermo Fisher Scientific).

### Transcriptomic Analyses

Raw reads from Ion Torrent PGM sequencing were quality filtered using CLC genomics workbench (CLC Genomic Workbench V8, CLC bio, Qiagen, Hilden, Germany) based on a minimum quality score of 0.05, a maximum sequencing length of 300 bp, and allowing for two ambiguous nucleotides. Filtered reads were then mapped to the *N. moscoviensis* genome (RefSeq: NZ_CP011801.1) using the mapping tool BBMap v35.92 developed by Bushnell B^1^. and counted using featureCounts (Liao et al., 2014) with the parameters “minid-0.95” with “fracOverlap-0.9” for a minimum alignment identity of 95% over 90% of the read length and “ambig = random” to assign reads with multiple top-scoring mapping locations randomly to a single location. In order to analyze the gene expression of the highly similar *nxrAB* paralogs and *nxrC* genes, a second mapping with more stringent conditions was performed. The alignment identity was increased to 100% (“minid-1.0”), and mapping was performed only for non-ambiguous reads (“ambig = toss”). Differential gene expression analysis was performed using the DESeq2 package (Love et al., 2014) in RStudio^2^. Differential gene expression over multiple time points was tested using the likelihood ratio test. Differential expression between time points was tested using the Wald test. Gene expression levels were compared by ranking the CDSs based on their reads per kilobase per million reads (RPKM) values, and the log_2_-fold to median was calculated. Circos was used for genome-wide visualizations of the gene expression levels (Krzywinski et al., 2009).

### DNA Extraction and Genome Sequencing

Biomass of 11 ml culture volume was harvested by centrifugation at 5250 × *g*, 4°C, 20 min. Genomic DNA was extracted using the MO BIO’s PowerSoil DNA Isolation Kit (Qiagen) according to the manufacturer’s protocol with the following modifications: Cell disruption was performed using a TissueLyser (TissueLyser LT, Qiagen) for two rounds of 30 s, with cooling of the sample on ice for 1 min. DNA quality was checked by agarose gel electrophoresis and subsequent staining using ethidium bromide.

In the following library preparations, all DNA quantification steps were performed using the Qubit dsDNA HS Assay Kit (Thermo Fisher Scientific) and all DNA purification steps were performed using AMPure XP beads (Beckman Coulter, Brea, CA, United States).

The DNA library for Illumina sequencing was constructed using the Nextera XT kit (Illumina, San Diego, CA, United States) with 5 μl of gDNA normalized to 0.2 ng/μl as input. The paired-end library was purified and quantified, and quality and size distribution were analyzed using the 2100 Bioanalyzer (Agilent Technologies). The library was sequenced on the Illumina Miseq platform using the MiSeq Reagent Kit v3 (Illumina) following the manufacturer’s protocol. The paired-end reads were trimmed, requiring a minimum read length of 100 bp and a quality limit of 0.01, and merged using CLC genomics workbench.

The DNA library for Nanopore sequencing was constructed using the Ligation Sequencing Kit 1D (SQK-LSK108, Oxford Nanopore Technologies, Oxford, United Kingdom) in combination with the Native barcoding Expansion Kit (EXP-NBD103, Oxford Nanopore Technologies) following the manufacturer’s protocol with 3 μg of gDNA as input. Shearing of the DNA was performed using the Covaris g-TUBE (Covaris Inc., Woburn, MA, United States) at 5,000 rpm for 60 s according to the manufacturer’s protocol. After shearing, a DNA repair treatment was performed using the NEBNext^^®^^ FFPE DNA Repair Mix (New England Biolabs, Ipswich, MA, United States) followed by a DNA purification step. End repair and dA-tailing was done using the NEBNext^^®^^ Ultra^TM^ II End Repair/dA-Tailing Module (New England Biolabs), followed by a DNA purification step. Selected barcodes for each sample were ligated using the Blunt/TA Ligase Master Mix (New England Biolabs) and again followed by a DNA purification step. Thereafter, adapters were ligated using the NEBNext^^®^^ Quick Ligation Module (New England Biolabs). The final library was purified and quantified and subsequently sequenced on the MinION sequencing device (Oxford Nanopore Technologies) according to the manufacturer’s protocol. For base calling, Albacore (Oxford Nanopore Technologies) was used.

The NanoPore and Illumina reads were separately mapped to the genome of *N. moscoviensis* (NZ_CP011801.1) using BBmap with the parameters “minid-0.8” and “ambig = random” for NanoPore reads and “minid-0.95” and “ambig = random” for Illumina reads. The mapping results were visualized using Circos (Krzywinski et al., 2009).

### Prediction Tools

The tool Phobius^3^ (Käll et al., 2004) was employed for the prediction of transmembrane helices and signal peptides in proteins.

## Results

### Culturing *N. moscoviensis* in a Continuous Stirred Tank Reactor

**FIGURE 1:**
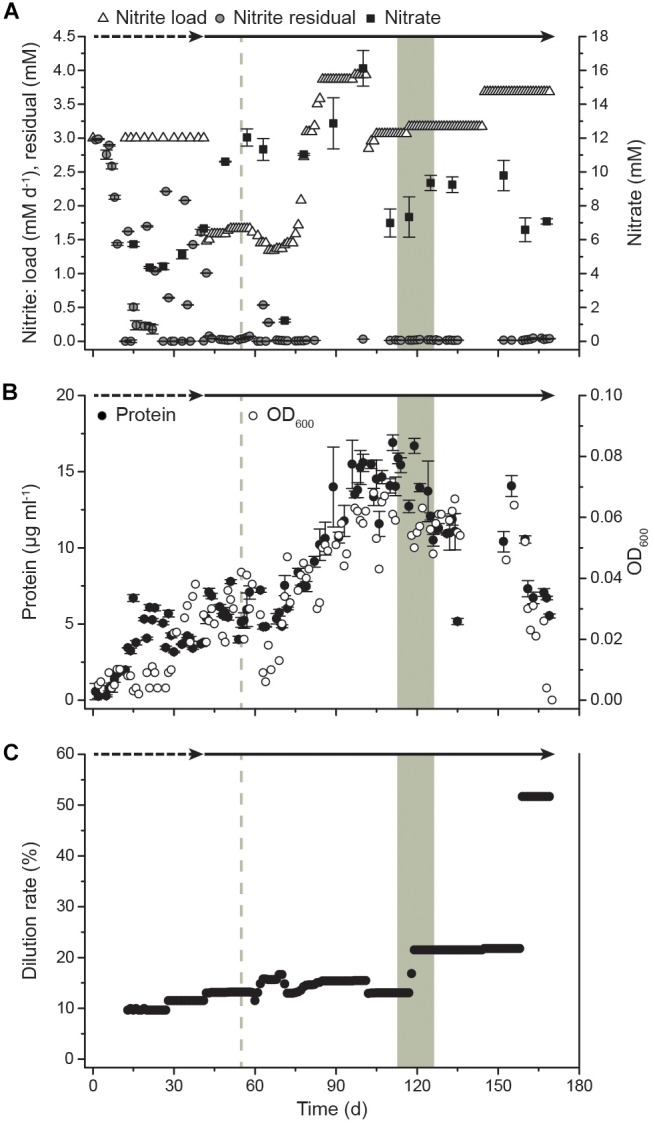
Growth of *N. moscoviensis* in the CSTR system. **(A)** Nitrite load, residual nitrite, and nitrate concentrations in the reactor. In the starting period, nitrite was manually supplied at 3 mM and replenished when consumed completely (timespan indicated by dashed arrow). After 41 days, nitrite was supplied continuously (timespan indicated by solid arrow). Error bars indicate standard deviation of three technical replicates. **(B)** Cellular growth, as indicated by OD_600_ measurements and protein content; error bars indicate standard deviation of three technical replicates. **(C)** Medium dilution rate. With increasing cell densities, the dilution rate was raised. The gray shading indicates the sampling period for transcriptomics (T1, day 113; T2, day 120; and T3, day 127), the gray dashed line indicates a pH increase to 9.2 (day 54) due to a technical failure.

After inoculation of the bioreactor, the culture immediately started to oxidize nitrite and grew without a lag phase (Figure 1). Initially, the bioreactor was operated as a semi-batch reactor by supplying pulses of nitrite to a final concentration of 3 mM to allow adaptation to the applied culturing conditions. After 41 days, the reactor system was switched to continuous nitrite feeding and operated as a CSTR. On day 54, the pH increased to 9.2 due to a technical failure, resulting in an increased residual nitrite concentration of 0.54 mM and a drop of biomass concentration in the following days. After this, the biomass gradually increased in the system with a maximum OD of 0.068 on day 103 and a maximum protein content of 16.9 μg ml^-1^ on day 111, corresponding to 1.17 × 10^6^ cells ml^-1^. Although OD seemed to be more strongly impacted by the pH perturbation event, overall OD and protein measurements showed the same trends. Dilution rates were applied ranging from 10% to 22% for continuous biomass harvesting. The maximum dilution rate corresponded to a mean cell retention time of 4.6 days (Figure 1). After a startup period of 12 days without removal of cells, the reactor started yielding a constant harvest of biomass of 13 mg protein per week in the effluent, which increased to a maximum weekly yield of >84 mg protein when the dilution rate was 22%. Samples for transcriptome analysis were taken over 2 weeks (days 113, 120, and 127), spanning three generations of cells. The daily supplied nitrite concentration was 3.1 ± 0.1 mM with residual concentrations of 0.2 ± 0.1 mM. Together with an OD of 0.057 ± 0.005, this indicated constant growth conditions during the sampling period. During this period, the protein yield in the reactor was 509 ± 355 μg protein mmol^-1^ nitrite and the specific growth rate μ was 0.14 ± 0.10 day^-1^. After 159 days, biofilm formation was observed. The biofilm quickly grew on the submerged metal and glass surfaces and planktonic growth decreased drastically as indicated by a drop in OD. This switch to growth in biofilm could not be reversed by applying a higher dilution rate (51.7%) to wash out quorum sensing molecules and other potential regulatory or inhibitory exometabolites. After day 169, planktonic cells could no longer be detected via OD measurements due to biofilm formation. Nitrite oxidation activity, however, remained high.

### Global Gene Expression Profile Under Continuous Growth

**FIGURE 2:**
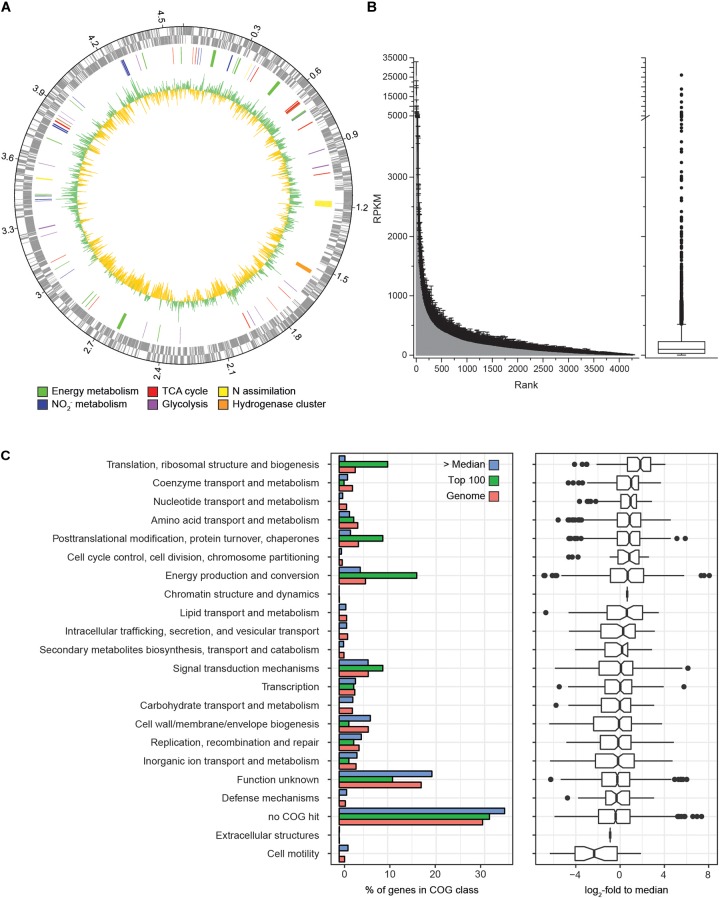
Genome-wide gene expression profile of *N. moscoviensis* during growth on nitrite. **(A)** From outside to inside, the rings show CDS in forward and reverse direction, selected CDS colored by metabolic categories and average gene expression levels (*n* = 3, with three technical replicates each) in log_2_-fold to median, with green representing transcription above and yellow below the median. **(B)** Average gene expression of all 4391 CDS in RPKM over three time points, with unidirectional error bars indicating standard deviation of three technical replicates. The median, 25th, and 75th percentiles are visualized in a box plot, with whiskers presenting 1.5 times the interquartile range. **(C)** Gene expression profiles sorted by COG functional categories. The left panel shows the percentage of genes falling within each category for all genes (red bars), among the 100 most highly transcribed CDS (green bars) and among all CDS expressed above median (blue bars). The right panel shows the expression data in log_2_-fold to median with 25th and 75th percentiles visualized in a box plot with whiskers presenting 1.5 times the interquartile range.

**FIGURE 3:**
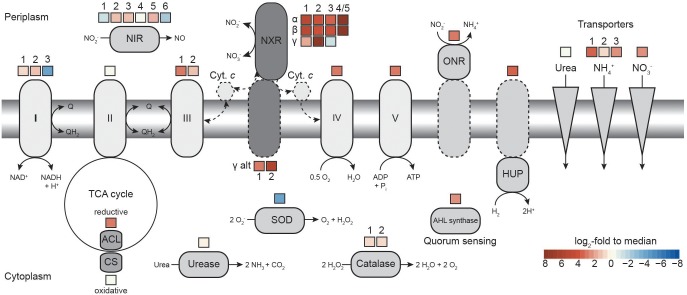
Comparison of transcription levels of selected metabolic features in *N. moscoviensis*. The expression level in log_2_-fold to median is indicated for each gene copy encoding the respective enzyme, for multi-subunit enzyme complexes average expression levels are shown. Dashed lines indicate putative features. Respiratory complexes are labeled in roman numerals. ACL, ATP-citrate lyase; AHL, acyl-homoserine-lactone; CS, citrate synthase; Cyt., cytochrome; HUP, group 2a [NiFe] hydrogenase; NIR, NO-forming nitrite reductase; NXR, nitrite oxidoreductase; ONR, octaheme nitrite reductase; and SOD, superoxide dismutase. For further details, please refer to the text and Supplementary Table S1.

Transcriptome analysis was performed to identify the gene expression profile of *N. moscoviensis* during continuous cultivation under stable nitrite-oxidizing conditions. In total, 509,685 mRNA reads from three time points with three replicates each were obtained and mapped to the 4391 predicted protein coding sequences (CDS) in the genome of *N. moscoviensis* (NZ_CP011801.1, Koch et al., 2015). Despite the resequencing of the genome (see below), this genome version was used for mapping, since it had higher coverage in Illumina sequencing and allows better comparability to previous publications and existing protein database entries. Time course differential gene expression analysis showed significant changes for only a very small CDS fraction (<0.5%, Table 1; for more details, see Supplementary Table S1), suggesting stable gene expression during continuous cultivation. Thus, the average of all three time points was used to calculate the global gene expression profile of *N. moscoviensis* (Figure 2A). Gene expression levels of all CDS were ranked by RPKM and compared by calculating the log_2_-fold change in RPKM relative to the median gene expression level (97.6 RPKM, Figure 2B). The large majority of genes were expressed; only 124 CDS had an RPKM of zero. This is in line with the high percentage of expressed genes in other chemolithotrophically grown AOB and NOB pure cultures analyzed in chemostat systems (Pérez et al., 2014). For an overview of dominant metabolic functions during constant growth, genes were grouped according to Clusters of Orthologous Groups (COG) categories (Figure 2C). Notably, many highly expressed genes could not be assigned to any COG category. Categories enriched in highly expressed genes included “Energy production and conversion,” “Translation, ribosomal structure and biogenesis,” and “Posttranslational modification, protein turnover, chaperones.” As detailed below, in-depth analysis was conducted for key metabolic features of *Nitrospira*, including nitrogen metabolism, respiratory chain, and CO_2_ fixation (Figure 3).

**Table 1:** Comparison of differential gene expression over the time course and between time points.

Number of genes	T1 vs. T2	T1 vs. T3	T2 vs. T3	Time course
Differentially expressed^1^	7	7	9	21
Non-differentially expressed	4384	4384	4382	4370


*^*1*^*p*_*adj*_ < 0.05.*

### Nitrogen Metabolism and Genome Resequencing

During continuous cultivation in the CSTR, the most highly expressed genes based on mapping with 95% alignment identity were *nxrA* (NITMOv2_4538), *nxrC* (NITMOv2_3624), and *nxrB* (NITMOv2_4533) (log_2_-fold to median 8.1, 7.6, and 7.3, respectively), together with a conserved exported protein of unknown function (NITMOv2_2496, Supplementary Table S1), demonstrating the key role of the NXR in the energy metabolism of nitrite-oxidizing *Nitrospira*. In order to distinguish among the highly similar *nxrA* and *nxrB* paralogs, an additional more stringent mapping (100% alignment identity and counting only unambiguous reads) was performed. In the following, only these mapping results are referred to when describing gene expression levels of *nxrA, nxrB*, and *nxrC* (Table 2).

**Table 2:** Gene expression levels of all putative nitrite oxidoreductase subunits encoded in the genome.

Gene name	Product	Notes^1^	kDa^2^	Identifier MicroScope	Identifier RefSeq	RPKM^3^	RPKM std. dev.
*nxrB_4/5*^4^	NXR beta subunit 4/5		49.9	NITMOv2_4533/ 4537	NITMOv2_RS20840/ RS20860	18,744	5212
*nxrA_5*	NXR alpha subunit 5		131.8	NITMOv2_4538	NITMOv2_RS20860	13,027	4869
*nxrA_1*	NXR alpha subunit 1		131.8	NITMOv2_0255	NITMOv2_RS01130	486	215
*nxrB_1*	NXR beta subunit 1		49.8	NITMOv2_0254	NITMOv2_RS01125	311	179
*nxrA_2*	NXR alpha subunit 2		131.6	NITMOv2_4028	NITMOv2_RS18515	179	76
*nxrB_2*	NXR beta subunit 2		49.8	NITMOv2_4027	NITMOv2_RS18510	59	46
*nxrA_3*	NXR alpha subunit 3		131.9	NITMOv2_4033	NITMOv2_RS18535	19	7
*nxrB_3*	NXR beta subunit 3		49.8	NITMOv2_4032	NITMOv2_RS18530	9	11
*nxrC_2*	NXR gamma subunit 2	1 SP	34.2/30.5	NITMOv2_3624	NITMOv2_RS16660	20,365	2508
*nxrC_1*	NXR gamma subunit 1	1 SP	30.8/27.4	NITMOv2_3617	NITMOv2_RS16630	354	115
*nxrC_3*	NXR gamma subunit 3	1 SP	28.7/26.6	NITMOv2_4208	NITMOv2_RS19340	26	36
*alt_nxrC_2*	NXR alternative gamma subunit 2	1 SP, 1 TMH, diheme cyt. *c*	66.8/63.8	NITMOv2_3640	NITMOv2_RS16730	5057	1073
*alt_nxrC_1*	NXR alternative gamma subunit 1	1 SP, 1 TMH	30.5/27.1	NITMOv2_0740	NITMOv2_RS03435	802	186


*^*1*^SP, signal peptide (Phobius prediction); TMH, transmembrane helix (Phobius prediction); cyt. *c*, cytochrome *c.*^*2*^If applicable, the molecular weight including/without the signal peptide is given. ^*3*^Mapping with 100% identity over 90% of the read length, only non-ambiguous reads are counted. ^*4*^Fused *nxrB* copy after genome deletion event, reads mapping against both gene copies were summed.*

The stringent mapping revealed a lack of reads mapping to the genomic region between locus tags NITMOv2_4533-4537, indicating a recombination between two *nxrB* paralogs, which resulted in the deletion of one copy of *nxrAB* and a transcriptional regulator (Supplementary Figure S1). This region was manually curated in the reference genome (Koch et al., 2015) and an erroneous assembly is therefore unlikely. Resequencing of the genome from the bioreactor culture confirmed the fused *nxrB* gene (*nxrB_4/5*) and the deletion of *nxrA_4* and the Sigma 54-dependent transcriptional regulator NITMOv2_4536. The fused *nxrB_4/5* gene and *nxrA_5* constituted the most highly expressed *nxrAB* paralog (18,744 and 13,027 RPKM, respectively; Table 2).

In addition, *N. moscoviensis* possesses genes for five putative NXR gamma subunits (Table 2) with predicted N-terminal signal peptides for translocation into the periplasm via the Sec pathway. The two alternative NxrC-like proteins (alt_NxrC_1 and 2) additionally contain a C-terminal transmembrane helix, indicating a membrane-associated periplasmic localization. Contrastingly, the lack of transmembrane helices in the remaining three NxrC candidates suggests that they are soluble periplasmic proteins.

Among the five putative NXR gamma subunits, *nxrC_2* showed by far the highest gene expression level, with a transcription level similar to the most highly transcribed *nxrAB* paralog (*nxrA_5* and *nxrB_4/5*), suggesting that the NXR complex of *Nitrospira* contains a soluble gamma subunit with a predicted mass of 30.5 kDa. The two membrane anchor candidates (*alt_nxrC_1* and *alt_nxrC_2*) were also highly transcribed, but with 25- and 4-fold lower expression levels, respectively. This indicates that these are not subunits of the NXR core complex; however, a role as anchor point for the NXR to channel electrons into the membrane-bound respiratory chain is conceivable. Alt_NxrC_2 clusters with two putative cytochrome *c* proteins and a putative cytochrome *bd*-like oxidase in the genome, forming a gene cluster that is highly conserved also in the phylogenetically distinct *Nitrospina* and anaerobic ammonium-oxidizing (anammox) bacteria, both of which also contain a NXR similar to *Nitrospira* and oxidize nitrite to nitrate (Lücker et al., 2010, 2013; de Almeida et al., 2011). Thus, it is tempting to speculate on an involvement of especially alt_NxrC_2 in nitrite oxidation, but its exact role remains to be determined.

**FIGURE 4:**
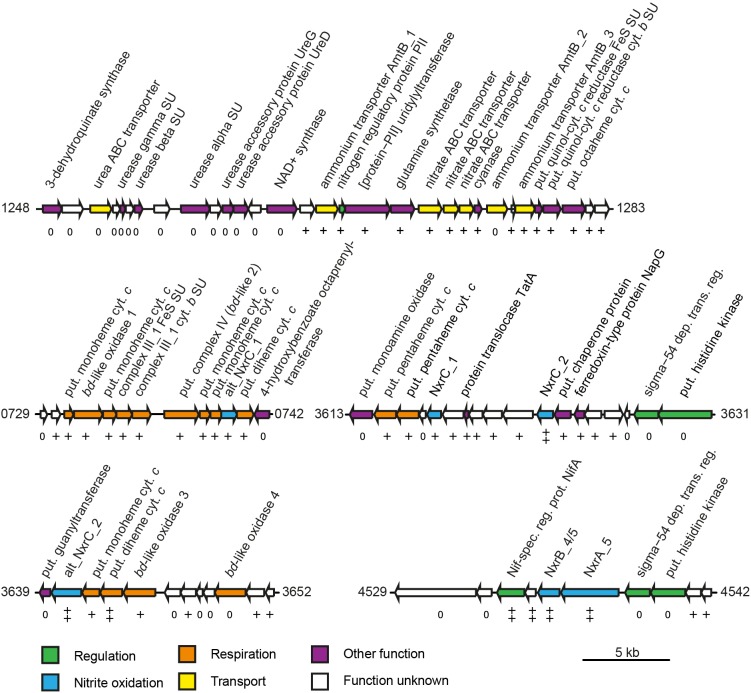
Schematic structure of selected highly expressed regions of the *N. moscoviensis* genome. Arrows represent gene length and orientation; the MicroScope gene identifier is given for the first and last gene of each region. The *nxrAB* 4/5 region is shown excluding the deletion described in this study (see Supplementary Figure S1). Gene transcription classes are based on log_2_-fold to median expression levels; all genes shown are within the top three pentiles (++, first pentile; +, second pentile; 0, third pentile). Cyt., cytochrome; dep. trans. reg., dependent transcriptional regulator; put., putative; spec. reg. prot., specific regulatory protein; and SU, subunit. For further details, please refer to Supplementary Table S1.

Besides genes encoding the NXR complex, many genes potentially involved in NXR maturation and regulation were also among the most highly transcribed genes. These included a putative TorD-like chaperone (NITMOv2_3625) that is encoded directly downstream of *nxrC_2* and potentially aids in NXR assembly (Lücker et al., 2010; Supplementary Table S2). In *N. moscoviensis*, at least two different regulation systems of NXR expression are present (Koch et al., 2015). Notably, the NifA-like transcriptional regulator gene (NITMOv2_4531) located upstream of the most highly expressed *nxrAB* paralog was on rank 14 in the transcriptome, whereas the sigma-54-dependent regulator (NITMOv2_4539) downstream showed much lower transcription levels (Figure 4). Homologs of this sigma-54-dependent regulator (NITMOv2_4539), but not of the NifA-like regulator, are encoded upstream of *nxrAB*_2 and *nxrAB*_3. These regulators had low gene expression levels consistent with the low gene expression levels of *nxrAB*_2 and *nxrAB*_3 (Table 2 and Supplementary Table S2). In the genomic vicinity of *nxrAB*_1, no transcriptional regulator could be identified, similar to the *nxrAB* locus in the closed genome of the complete nitrifier *N. inopinata* (Daims et al., 2015).

Several putative copper-containing dissimilatory nitrite reductases (NirK) are encoded in the genome. Of these, one complete (NirK_1, NirK_6) and one partial paralog (NirK_2, NirK_5) are present in two identical copies each. Of all NirK encoding genes, only *nirK*_3 and the duplicated partial *nirK* genes (*nirK_2, nirK_5*) showed transcription levels above median gene expression (Figure 3).

Most genes associated with nitrogen transport, acquisition, assimilation, and its regulation are clustered in one genomic region in *N. moscoviensis* (Figure 4). Within this locus, subunits of a urea ABC transporter, urease subunits, and urease accessory proteins showed moderate transcription, indicating constitutive expression of the urea metabolic genes even in the absence of urea. In contrast, the other genes of this region showed strong transcription with the exception of one of the three ammonium transporter paralog genes (*amtB_2*), which was transcribed considerably less than the two other AMT-type transporters. Notably, the lowest and highest expressed ammonium transporters AmtB_2 and AmtB_1, respectively, showed a high amino acid similarity (74%), whereas AmtB_3 is less similar to these (44–46%), but was highly expressed. The cyanate hydrolyzing cyanase was among the 100 most highly expressed genes, despite the lack of external cyanate. The only supplied N source in the medium was nitrite, which must be reduced to ammonium for assimilation. Consistent with the need for assimilatory nitrite reduction, the octaheme nitrite reductase (ONR), which was previously identified in the genomes of *N. moscoviensis* and *N. japonica* (Koch et al., 2015; Ushiki et al., 2018), together with two upstream genes encoding a Rieske/cytochrome *b* complex showed high expression levels (Figure 4).

### Respiration and Carbon Fixation

Previous genome studies revealed that canonical *Nitrospira* possess multiple copies of several complexes of the respiratory chain (Lücker et al., 2010; Koch et al., 2015; Ushiki et al., 2018). The genome of *N. moscoviensis* contains three distinct copies of complex I, two of complex III, two cytochrome *bd* oxidases, and four copies of a putative cytochrome *bd*-like oxidase, which all show limited partial similarity to cytochrome *bd* oxidase subunit I (Supplementary Table S2). Based on the presence of putative heme *b* and copper binding sites characteristic for heme-copper cytochrome *c* oxidases, one of these *bd*-like oxidases (*bd*-like_2) was suggested to function as a novel terminal oxidase (Lücker et al., 2010). NADH-quinone oxidoreductase 1 (CI_1) was expressed above median level (Figure 3). It belongs to clade 3 of the recently described 2M complex I type (Chadwick et al., 2018) and contains the characteristic duplication of NuoM, but lacks genes for NuoE, NuoF, and NuoH. NADH-quinone oxidoreductase 2 (CI_2) and 3 (CI_3) resemble canonical complex I and both contain fused NuoCD subunits. CI_2 had the highest gene expression levels of the three paralogous complexes, whereas CI_3, which also is not conserved in most other *Nitrospira*, was hardly transcribed (Figure 3). *N. moscoviensis* furthermore encodes additional non-operonal copies of putative NADH-quinone oxidoreductase subunits, of which the *nuoM* and *nuoL* genes were lowly expressed, while a single *nuoF* gene showed high expression levels 2.5 log_2_-fold above median and was thus the most highly transcribed complex I gene (Supplementary Table S2).

The gene cluster for complex II (succinate dehydrogenase/fumarate reductase; SDH/FRD) showed gene expression below the median. In addition, a gene (NITMOv2_0695) coding for a flavoprotein subunit annotated as either SdhA of complex II or NadB of a L-aspartate oxidase is located elsewhere in the genome. The expression of this single gene was much higher than the genes for SDH/FRD and the canonical L-aspartate oxidase (Supplementary Table S2).

Besides the Rieske/cytochrome *b* complex putatively involved in assimilatory nitrite reduction, *N. moscoviensis* encodes two copies of complex III, which both displayed above-median gene expression levels. The gene cluster with the higher expression (CIII_1) contains the complex III core genes for a Rieske iron–sulfur and a cytochrome *b* subunit and is encoded in a genomic location encoding for several cytochrome *c* proteins (see below). The second cluster (CIII_2) contains genes for the Rieske, a fused cytochrome *b*/*c* and an additional diheme cytochrome *c* subunit. While the Rieske subunit of CIII_2 displayed very high gene expression in a similar range to CIII_1, the cytochrome *c* and fused cytochrome *b*/*c* subunits were only moderately expressed. Interestingly, CIII_1 is localized in a genomic region of consistent high gene expression levels, together with the putative terminal oxidase (*bd*-like_2, CIV), an additional *bd*-like oxidase (*bd*-like_1) and one of the two alternative NXR gamma subunit candidates (alt_nxrC_1). In addition, several strongly expressed cytochrome *c* proteins are encoded in this genomic region (Figure 4). Together, these proteins may form a respiratory supercomplex comprising of complexes III and IV, which furthermore might be able to directly interact with the soluble NXR complex via the alternative NXR gamma subunit. Similarly, one of the remaining two *bd*-like oxidases (*bd*-like_3) also forms a gene cluster with an alternative NXR gamma subunit candidate (alt_NxrC_2) and two *c*-type cytochromes, possibly forming a complex involved in reverse electron flow for assimilatory processes. These genes were among the 40 strongest transcribed genes, supporting their important central role for growth by nitrite oxidation.

The genome encodes numerous additional cytochrome *c* proteins that might be involved in electron transport. The monoheme cytochrome *c* protein NITMOv2_3685 showed the highest expression level and was ranked as the 12^th^ most highly transcribed gene of *N. moscoviensis* (Supplementary Table S1). In addition to the *bd*-like oxidases mentioned above, the genome also contains two copies of a canonical heterodimeric cytochrome *bd* oxidase (CydAB_1 and CydAB_2). Consistent with its proposed function of receiving electrons from low potential donors via the quinone pool (Lücker et al., 2010), both copies were very weakly expressed under nitrite-oxidizing conditions (Supplementary Table S2). Complex V of the respiratory chain, the F_1_F_o_-type ATP synthase, showed strong to very strong expression for both the membrane-integral F_o_ and the catalytic F_1_ sector, which are encoded in separate gene clusters in the *N. moscoviensis* genome.

Genes of the TCA cycle showed high variation in expression levels, ranging from below median to very high, corresponding to their involvement in either the reductive or the oxidative direction of the cycle. Consistent with autotrophic growth, key genes of the rTCA cycle for CO_2_ fixation were highly expressed (Supplementary Table S2), including pyruvate:ferredoxin oxidoreductase (POR), 2-oxoglutarate:ferredoxin oxidoreductase (OGOR), and ATP-citrate lyase. *N. moscoviensis* also encodes a citrate synthase; this enzyme was previously assumed to be exclusively involved in the oxidative TCA (oTCA) cycle, but recently was demonstrated to also participate in the reductive direction of the cycle by cleaving citrate (Mall et al., 2018; Nunoura et al., 2018). However, the presence of an ATP-citrate lyase and the low expression level of this citrate synthase suggest a canonical function in the oTCA cycle. Additionally, the low expression levels of the NADH-dependent 2-oxoglutarate and pyruvate dehydrogenase complexes indicate the expected minor role of the oTCA cycle in the autotrophic metabolism of *Nitrospira*.

### Expression of Other Metabolic Key Features

Aside from nitrite, *N. moscoviensis* can also use hydrogen as electron donor for aerobic respiration (Koch et al., 2014). Although nitrite-oxidizing conditions were applied, three neighboring genes in the hydrogenase-containing *hup* locus were strongly transcribed. The small and large subunit of the group 2a [NiFe] hydrogenase and a conserved protein of unknown function (NITMOv2_1641) were ranked among the 300 most highly transcribed genes. In contrast, the expression of the remaining *hup* locus, involved in regulation and maturation of the hydrogenase holoenzyme, ranged from 0.8 below to 1.3 log_2_-fold above median (Supplementary Table S2). This constitutive expression of the hydrogenase structural genes under nitrite-oxidizing conditions has been previously reported and was hypothesized to function as a possible H_2_ sensing mechanism (Koch et al., 2014).

*N. moscoviensis* possesses several oxygen defense mechanisms, including catalase and superoxide dismutase and other proteins potentially involved in protection against reactive oxygen species (ROS). While the two identical catalase gene copies were moderately expressed, the superoxide dismutase was among the lowest expressed genes (Figure 3). Additional putative ROS defense proteins include several bacterioferritin and peroxidase, which have been proposed to function as H_2_O_2_ scavengers and protect against ROS (Lücker et al., 2010). The two neighboring bacterioferritin genes (NITMOv2_0876/7) were among the 300 most highly expressed genes, while an additional putative bacterioferritin gene (NITMOv2_2541) showed weak expression (1.9 log_2_-fold below median). Similarly, transcription levels of the different peroxidase genes varied widely from weak to strong expression (Supplementary Table S1).

Recently, *N*-acyl-L-homoserine lactone (AHL)-based quorum sensing systems have been identified in several *Nitrospira* genomes, including *N. moscoviensis* (Mellbye et al., 2017; Ushiki et al., 2018). The quorum sensing systems of *Nitrospira* rely on the interplay of the autoinducer AHL, produced by AHL synthase, and a LuxR-type transcriptional regulator that triggers the expression of other quorum sensing regulated genes. The AHL synthetase (Mellbye et al., 2017) showed a gene expression level of 2.2 and the associated LuxR-like transcriptional regulator of 2.8 log_2_-fold above median (Supplementary Table S2), suggesting production of quorum sensing molecules and the potential for cell-to-cell communication in the culture.

## Discussion

### Cultivation in a CSTR

Culturing microorganisms in CSTRs has many advantages compared to batch cultivation. Reactor systems allow for precise control of key parameters such as substrate supply, pH, temperature, and oxygen concentrations. In addition, continuous flow of medium prevents not only a lack of substrate and trace elements, but also the accumulation of potential inhibitory metabolites, and allows a precise control of growth rates (see, for example, Weusthuis et al., 1994). The application of CSTRs is especially advantageous to cultivate NOB, because maintaining and cultivating members of this functional group is laborious and time-consuming (Spieck and Lipski, 2010). Since nitrite is inhibitory and also toxic at higher concentrations, our setup used continuous nitrite feeding, which avoided both starvation periods and elevated nitrite concentrations. During the transcriptome sampling period, the growth rate corresponds to a generation time of 5 days, which is higher than the generation times of 0.5 and 1.3 days at maximum growth reported previously for *N. moscoviensis* (Ehrich et al., 1995; Nowka et al., 2015). In line with the trend between previous reports of growth rates and yield of *N. moscoviensis* (Ehrich et al., 1995; Nowka et al., 2015), with a lower growth rate, we observed a higher growth yield. The low growth rate indicates limiting conditions, which potentially were caused by the operation of the reactor at low residual nitrite concentrations below the optimum concentration of 0.35 mM nitrite (Ehrich et al., 1995). It also demonstrates that faster growth of the culture could be obtained in future experiments by optimizing the operational settings of the reactor to a dilution rate closer to the maximum growth rate of the culture.

The successful operation of the CSTR system for over 5 months, the continuous production of biomass, and the constant gene expression profile over several generations during a timeframe of 2 weeks demonstrated this approach to be a valuable tool for the cultivation of *Nitrospira*. However, the culture slowly switched to growth in biofilm after approximately 159 days, and our attempt to wash out potential biofilm-inducing signaling molecules from the culture by increasing media flow rates failed to revert the culture back to planktonic growth. To avoid biofilm formation, future cultivation strategies could include a membrane bioreactor setup with high medium flow rates, which would allow the constant washing out of signaling molecules from the culture. Still, the application of bioreactors can greatly improve the culturing procedure of *Nitrospira* and other slow-growing NOB in terms of stability of culturing conditions and effort needed for culture maintenance. In addition, continuous cultivation strategies using bioreactors can be used to upscale biomass production. This will facilitate biomass intensive proteomic and biochemical characterizations, which are key to further expand our understanding of the *Nitrospira* metabolism (Lancaster et al., 2018).

### NXR Composition of *N. moscoviensis*

Under nitrite-oxidizing conditions, among the most highly expressed genes were *nxrA, nxrB*, genes encoding a soluble NXR gamma subunit candidate (*nxrC_2*), as well as a potentially NXR-specific transcriptional regulator. This consolidates the key role of the NXR in the autotrophic metabolism of *Nitrospira*. Notably, it furthermore suggests a functional role of a presumably soluble gamma subunit within the NXR complex, as NxrC_2 has no predicted transmembrane helix. The predicted mass of this protein (30.5 kDa) is close to the apparent mass reported for one of three unidentified protein bands (62 and 29 kDa) of the nitrite-oxidizing particles purified by Spieck and coworkers (Spieck et al., 1998; Lücker et al., 2010). However, the *Nitrospira* NXR was previously reported to be membrane associated (Spieck et al., 1998), which would necessitate interaction with a membrane anchor subunit or a membrane-bound complex. Proteins that might serve as a membrane anchor for the NXR complex are the alternative NxrC candidates, as was suggested for *Nitrospina gracilis* (Lücker et al., 2013). One of these alternative *nxrC* (*alt_nxrC_2*) is highly expressed and encodes a protein containing a transmembrane helix and two heme-binding motifs. On the one hand, its expression level is 2.5- to 4-fold lower than the other NXR subunits including *nxrC_2*, making a direct interaction with the NXR complex questionable. On the other hand, it is the only NXR subunit candidate whose mass is in range of the reported nitrite-oxidizing system protein band of 62 kDa (Spieck et al., 1998). This alternative NxrC candidate forms a highly transcribed gene cluster with two membrane-bound *c*-type cytochromes and a *bd*-like oxidase (*bd*-like_3), which furthermore is duplicated a few genes downstream in all *Nitrospira* genomes (*bd*-like_4). While the metabolic role of this putative membrane complex remains to be determined, it has been found to be highly conserved in anammox bacteria and *N. gracilis* (Lücker et al., 2010, 2013; de Almeida et al., 2011), indicating involvement in nitrite oxidation, possibly by acting as anchor point for the NXR complex and putatively allowing interaction with the quinone pool for reverse electron transport (Kartal et al., 2013). Notably, the gene for the second membrane-associated alternative NxrC (*alt_nxrC_1*) was also highly expressed, but six fold less than *alt_nxrC_2*. Differences in genomic neighborhood and protein domains (Table 2 and Supplementary Table S1) hint at a functional differentiation of the two alternative NxrC paralogs. It is tempting to speculate that alt_NxrC_1 interacts with the terminal oxidase, since its gene is located in close vicinity to the putative complex IV (*bd*-like_2). In addition, the two strongly expressed cytochrome *c* genes encoded in-between the genes for the putative terminal cytochrome *c* oxidase and alt_NxrC_1 might function as electron shuttles between the two proteins. Again, this alternative NxrC might interact with the NXR complex, this time facilitating direct electron transport from NXR to complex IV.

Despite the high similarity of the NxrAB paralogs in *N. moscoviensis* (Koch et al., 2015), the high expression of only one *nxrAB* paralog indicates a differential regulation of the *nxrAB* loci. Former genomic analyses suggested different transcriptional regulation mechanisms for the *nxrAB* paralogs in *N. moscoviensis* as well as in the closed genomes of *N. defluvii* and *N. japonica* (Lücker et al., 2010; Koch et al., 2015; Ushiki et al., 2018). These involve different sigma-54-dependent transcriptional regulators, with a NifA family protein being the most highly transcribed regulatory protein in *N. moscoviensis* under the constant cultivation conditions applied in this study. Future studies comparing gene expression profiles of *Nitrospira* incubated with various nitrite concentrations and under anoxic conditions using nitrate as electron acceptor may help to resolve the different roles of the NXR paralogs.

### Nitrite Reduction and Nitrogen Assimilation

*N. moscoviensis* contains several *nirK* genes, which are constitutively transcribed under nitrite-oxidizing conditions. However, the physiological role of these dissimilatory nitrite reductases in nitrite-oxidizing *Nitrospira* remains to be determined. A major role in denitrification seems to be unlikely, since stoichiometric conversion of nitrate to nitrite was observed, when *N. moscoviensis* was grown on formate with nitrate as alternative electron acceptor (Koch et al., 2015). In *Nitrobacter*, NO production was proposed to be involved in regulating the redox state of the cells by stimulating reverse electron flow from nitrite oxidation under low O_2_ conditions, mediated by NO reversible inhibiting the cytochrome oxidase (Starkenburg et al., 2008a). Potentially, NO might have a similar regulatory role of the cells redox state, if it can inhibit the yet to be characterized terminal oxidase of *Nitrospira*.

Nitrite was the only N source available in the CSTR and the cells thus had to reduce nitrite for assimilation. High gene expression levels of an ONR corroborates its suggested role in assimilatory nitrite reduction in *N. moscoviensis*, which lacks the assimilatory ferredoxin-nitrite reductase NirA found in other *Nitrospira* (Lücker et al., 2010; Koch et al., 2015, 2018; Sakoula et al., 2018; Ushiki et al., 2018). This role was recently confirmed in *N. japonica* by gene expression analysis, which expressed the ONR during growth on nitrite, but not when ammonia was added to the medium as N source (Ushiki et al., 2018). The similar expression levels of the genes for the ONR and the neighboring Rieske/cytochrome *b* subunits support the hypothesis that assimilatory nitrite reduction by the ONR is coupled to the quinone pool in *Nitrospira* (Koch et al., 2015). Consistent with the periplasmic localization of the ONR, two of three ammonium transporters encoded in the genome were strongly expressed. In ammonia-oxidizing archaea (AOA), two distinct copies of AMT-type transporters were expressed simultaneously in the presence of ammonium, while only one copy was constitutively expressed during ammonium starvation as a system to reinitiate growth (Qin et al., 2018). In the AOA *Candidatus* Nitrosopelagicus brevis, one AMT-type transporter was even among the top 10 highest transcribed genes during growth on urea as N source despite the absence of external ammonia (Carini et al., 2018). While the divergent expression levels of the AMT-type transporters in *N. moscoviensis* might hint at an adaption to different ammonium levels, the exact roles of the different AMT paralogs remain to be determined. Furthermore, the moderate expression of urease and strong expression of cyanase hint to an adaption to transient ammonium availability, allowing cells to use urea and cyanate as alternative ammonium sources (Koch et al., 2015; Palatinszky et al., 2015). An additional role of cyanase might be the detoxification of internally produced cyanate caused by spontaneous abiotic breakdown of carbamoyl phosphate, an important intermediate in the pyrimidine and arginine metabolism (Suzuki et al., 1996; Daims et al., 2016).

### Functional Differentiation Between Respiratory Complex Paralogs

The need to produce NADH as reduction equivalents for anabolic reactions during autotrophic growth demands reverse electron flow from nitrite to NAD^+^. The expression levels of the canonical complex I indicate that NADH-quinone oxidoreductase 2 rather than the homologous NADH-quinone oxidoreductase 3 catalyzes NADH production under nitrite-oxidizing conditions. However, future work is required to further investigate the potential functional differentiation of these two complex I copies by analyzing gene expression of complex I copies under growth conditions that favor electron flow in the oxidative direction of the respiratory chain.

In addition to two canonical complexes I, the genome contains a gene cluster for an additional NADH-quinone oxidoreductase (CI_1) that belongs to clade 3 of the recently described 2M complex I type. These complex I homologs are characterized by a lengthened NuoL and a second NuoM subunit thought to result in a higher stoichiometry of protons translocated per reaction cycle (Chadwick et al., 2018). It was hypothesized that the 2M complex might participate in reverse electron transport from quinol to the low potential electron carrier ferredoxin (Lücker et al., 2013; Chadwick et al., 2018). Reduced low potential ferredoxins are required for OGOR and POR, two key enzymes of the rTCA cycle for CO_2_ fixation during autotrophic growth. The high expression levels of genes involved in the rTCA cycle indicate the need for ferredoxin as electron carrier, which potentially might be reduced via the 2M-type complex I, since no other known ferredoxin reduction mechanisms could be identified in *Nitrospira* to date (Lücker et al., 2010). As described for other clade 3 operons of 2M-type complex I, the CI_1 gene cluster in *N. moscoviensis* lacks *nuoH, nuoE*, and *nuoF* genes and contains an enlarged *nuoG* homolog. Interestingly, a separate *nuoF* gene (NITMOv2_4064) together with genes encoding a ferredoxin and an iron–sulfur cluster insertion protein forms a highly expressed gene cluster. This NuoF might interact with Cl_1 and could be involved in the reduction of ferredoxin.

Expression levels of the two quinol–cytochrome *c* reductase paralogs showed a higher transcription of quinol–cytochrome *c* reductase 1 compared to the second complex III copy. Interestingly, the genes of this quinol–cytochrome *c* reductase cluster together with genes encoding the putative complex IV and the NXR membrane anchor gamma subunit candidate alt_NxrC_1. The same genomic region also encodes a second putative cytochrome *bd*-like oxidase (*bd*-like_1) with yet unknown role in the metabolism of *Nitrospira*, and several *c*-type cytochromes. All of these genes were strongly transcribed, indicating the need for orchestrated regulation. It is tempting to speculate that these proteins could form a respiratory supercomplex composed of complexes III and IV, which have been previously reported for several bacterial respiratory chains (Stroh et al., 2004). The formation of supercomplexes can convey many advantages such as substrate channeling, stabilization of the complexes, and prevention of electron leakage (Stroh et al., 2004; Genova et al., 2008; Winge, 2012).

In addition to the four paralogous copies of *bd*-like oxidases, *N. moscoviensis* contains two canonical heterodimeric cytochrome *bd* oxidases (CydAB_1 and CydAB_2) as additional, quinol-dependent terminal oxidases. Their proposed function is to receive electrons derived from low-potential electron donors via the quinone pool (Lücker et al., 2010). Consistent with the lack of low-potential electron donors in the cultivation medium, cytochrome *bd* oxidase 1 and 2 showed low expression levels.

## Conclusion

The transcriptomic analysis of *N. moscoviensis* cultivated in a CSTR system under constant, nitrite-oxidizing conditions gave vital insights into the energy metabolism of *Nitrospira*. The moderate expression of two different complex I copies during growth on nitrite supports the proposed functional differentiation between the canonical and the 2M type complex I and indicates a role of the latter in reducing ferredoxin. Furthermore, the expression pattern of NXR genes suggests the involvement of a soluble gamma subunit with a role in electron shuttling, and a possible involvement of the alternative NXR gamma subunit candidates as membrane anchors to facilitate interaction of the NXR with the respiratory chain. *N. moscoviensis* is a metabolically versatile organism, which has the ability to not only aerobically grow on nitrite but also use alternative substrates. This metabolic versatility must be reflected in the respiratory chain, since electrons derived from alternative donors have varied entry points into the respiratory chain and thus take different electrons transport routes and directions. Transcriptomic analysis confirmed differences in expression levels between multiple copies of several respiratory complexes, which indicate functional differentiation within the respiratory chain of *N. moscoviensis* as an adaption to its versatile lifestyle. It remains to be determined if functional differentiations between copies of the respiratory complexes are mainly linked to directionality or arise from specific interactions with other enzymes such as the NXR complex. Future comparative studies of *N. moscoviensis* grown on different substrates will help to deconvolute the differentiations and interactions among and between different respiratory complexes and the NXR. Stable cultivation and efficient large-scale biomass production in a bioreactor will greatly benefit the biochemical, proteomic, and complexomic analyses needed to completely resolve the respiratory chain in *Nitrospira*.

## Data Availability

NanoPore and Illumina raw reads were deposited at the NCBI Sequence Read Archive (SRA) under accession number PRJNA508504 (https://www.ncbi.nlm.nih.gov/sra/PRJNA508504). RNA-Seq data was deposited in the NCBI Gene Expression Omnibus (GEO) database repository (Edgar et al., 2002) under GEO Series accession number GSE123406 (https://www.ncbi.nlm.nih.gov/geo/query/acc.cgi?acc=GSE123406).

## Author Contributions

SL, AM, and MJ contributed to conception and design of the study. AM conducted the experiments. AM, CL, and HK carried out the data analysis. CL carried out the statistical analysis. AM, CL, HK, and SL wrote the manuscript. All authors contributed to revision of the manuscript, and read and approved the submitted version.

## Conflict of Interest Statement

The authors declare that the research was conducted in the absence of any commercial or financial relationships that could be construed as a potential conflict of interest.

## Footnotes

1 https://sourceforge.net/projects/bbmap/

2 https://www.rstudio.com/

3 http://phobius.sbc.su.se
