# Supplementary material for: Cultivation and Transcriptional Analysis of a Canonical Nitrospira Under Stable Growth Conditions
Source: Front Microbiol. 2019 Jun 26;10:1325. doi: 10.3389/fmicb.2019.01325 (PMC6606698; doi:10.3389/fmicb.2019.01325)
Supplement: Supplementary file 1 [file Data_Sheet_1.pdf]

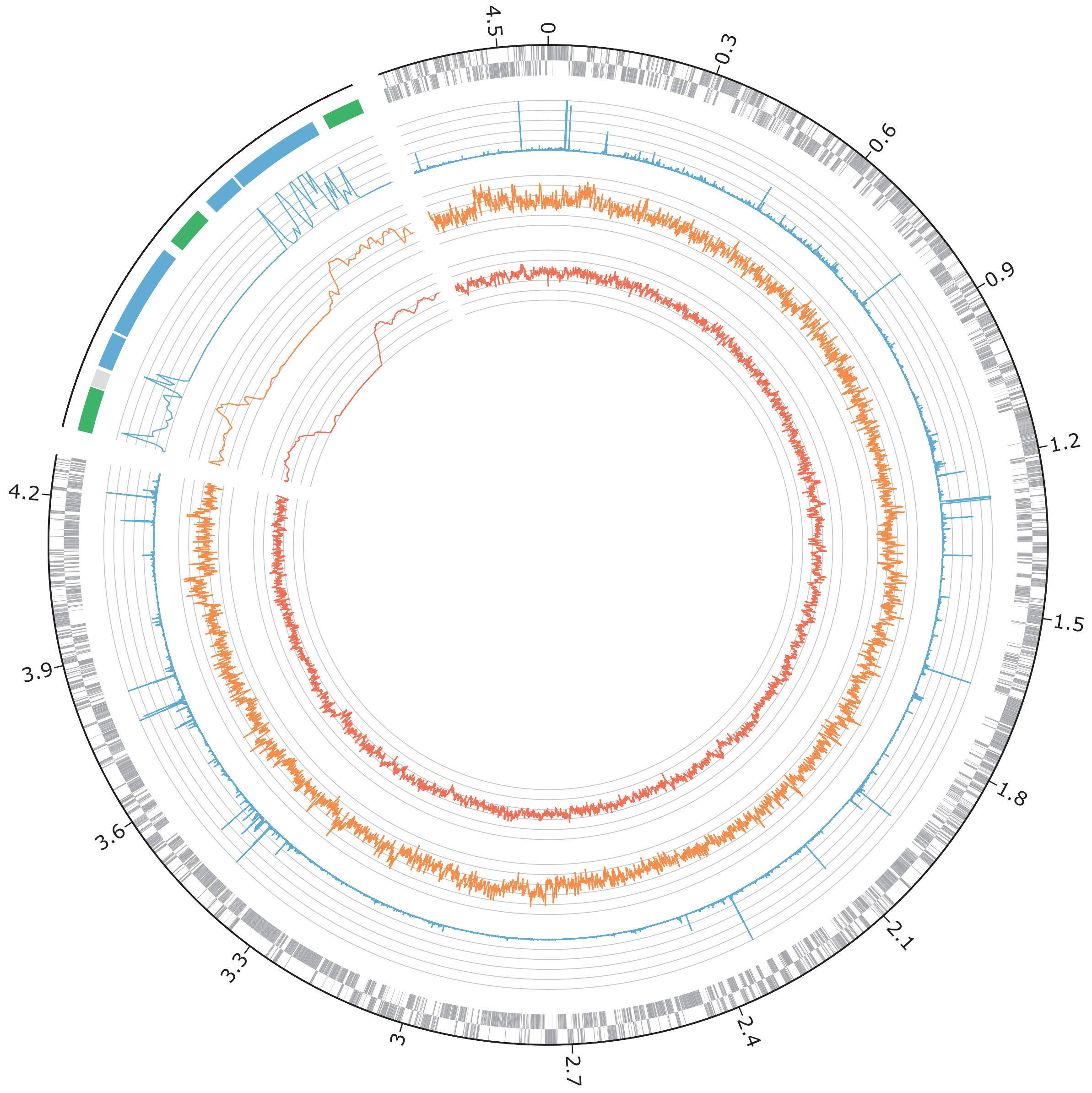

Supplementary figure S1. Deletion in the genome of *N. moscoviensis*. The outermost ring shows all CDS predicted in the published *N. moscoviensis* genome (NZ_CP011801.1). In the inner rings coverage of mapped reads is shown for RNA-Seq reads (blue) as well as for Illumina (orange) and NanoPore (red) reads obtained by resequencing of the genome. The zoomed region spans from NITMOv2_4531 – 4539 (NXR in blue, regulators in green, hypothetical protein in grey). For the genome resequencing data, the zoomed region shows a coverage window of 100 bp and for the remaining genome a coverage window of 1000 bp was used. For the RNA-Seq data, a coverage window of 100 bp is shown for the whole genome and the visualization of the highest coverage was limited to 100-fold.
